# Early effects of low dose bevacizumab treatment assessed by magnetic resonance imaging

**DOI:** 10.1186/s12885-015-1918-1

**Published:** 2015-11-14

**Authors:** Jon-Vidar Gaustad, Trude G. Simonsen, Ragnhild Smistad, Catherine S. Wegner, Lise Mari K. Andersen, Einar K. Rofstad

**Affiliations:** Group of Radiation Biology and Tumor Physiology, Department of Radiation Biology, Institute for Cancer Research, Oslo University Hospital, Oslo, Norway

**Keywords:** Bevacizumab, Antiangiogenic treatment, Blood perfusion, Intratumor heterogeneity, DCE-MRI, DW-MRI

## Abstract

**Background:**

Antiangiogenic treatments have been shown to increase blood perfusion and oxygenation in some experimental tumors, and to reduce blood perfusion and induce hypoxia in others. The purpose of this preclinical study was to investigate the potential of dynamic contrast enhanced magnetic resonance imaging (DCE-MRI) and diffusion weighted MRI (DW-MRI) in assessing early effects of low dose bevacizumab treatment, and to investigate intratumor heterogeneity in this effect.

**Methods:**

A-07 and R-18 human melanoma xenografts, showing high and low expression of VEGF-A, respectively, were used as tumor models. Untreated and bevacizumab-treated tumors were subjected to DCE-MRI and DW-MRI before treatment, and twice during a 7-days treatment period. Tumor images of *K*^trans^ (the volume transfer constant of Gd-DOTA) and *v*_e_ (the fractional distribution volume of Gd-DOTA) were produced by pharmacokinetic analysis of the DCE-MRI data, and tumor images of ADC (the apparent diffusion coefficient) were produced from DW-MRI data.

**Results:**

Untreated A-07 tumors showed higher *K*^trans^, *v*_e_, and ADC values than untreated R-18 tumors. Untreated tumors showed radial heterogeneity in *K*^trans^, i.e., *K*^trans^ was low in central tumor regions and increased gradually towards the tumor periphery. After the treatment, bevacizumab-treated A-07 tumors showed lower *K*^trans^ values than untreated A-07 tumors. Peripherial tumor regions showed substantial reductions in *K*^trans^, whereas little or no effect was seen in central regions. Consequently, the treatment altered the radial heterogeneity in *K*^trans^. In R-18 tumors, significant changes in *K*^trans^ were not observed. Treatment induced changes in tumor size, *v*_e_, and ADC were not seen in any of the tumor lines.

**Conclusions:**

Early effects of low dose bevacizumab treatment may be highly heterogeneous within tumors and can be detected with DCE-MRI.

**Electronic supplementary material:**

The online version of this article (doi:10.1186/s12885-015-1918-1) contains supplementary material, which is available to authorized users.

## Background

To grow beyond a few millimeters in size, solid tumors need to establish vascular networks that can supply the tumor cells with oxygen and other nutrients [1]. The tumor cells produce and secrete several proteins that stimulate or inhibit angiogenesis, and the rate of angiogenesis is governed by the balance between these pro- and antiangiogenic factors [2]. Several antiangiogenic strategies are being investigated, including treatments with endogenous antiangiogenic facors or small peptides that mimic these factors [3, 4], monoclonal antibodies against proangiogenic factors or their receptors [5, 6], and tyrosine kinase inhibitors which may target multiple proangiogenic receptors [7]. The antiangiogenic treatments are generally not cytotoxic, and treatment-induced reductions in tumor volume often appear late compared to vascular effects [8]. It is therefore recognized that assessment of functional parameters are needed to detect early effects of antiangiogenic treatment.

Although antiangiogenic treatments may inhibit tumor growth when used alone, the therapeutic benefit may be even greater when used in combination with conventional therapies such as radiation and chemotherapy [9]. The effect of radiation and chemotherapy can be significantly affected by the tumor microenvironment, thus tumors with extensive hypoxia are more resistant to radiation and some forms of chemotherapy, and poor blood perfusion may reduce the uptake of chemotherapeutic drugs [10]. Antiangiogenic treatments have been reported to reduce blood perfusion and induce hypoxia in some experimental tumors [6, 11], and to increase blood perfusion and oxygenation in others [5, 12]. The reasons for these different effects are not well understood but may have significant impact on combination therapy [9]. It has been suggested that the effect of antiangiogenic treatment may vary with time after treatment, and that low doses of the antiangiogenic agent are required to increase blood perfusion and oxygenation [13]. It is also possible that the effect of antiangiogenic treatment may vary within tumors, although studies investigating this possibility are sparse.

Dynamic contrast enhanced magnetic resonance imaging (DCE-MRI) and diffusion-weighted MRI (DW-MRI) have been used to evaluate the effect of antiangiogenic treatment [14]. In DCE-MRI, pharmacokinetic models are used to describe the tumor uptake of an intravenously administered contrast agent. The most common model is the generalized pharmacokinetic model of Tofts et al. [15]. In this model, the transfer rate constant, *K*^trans^, and the fractional distribution volume, *v*_e_, are estimated. *K*^trans^ generally reflects blood perfusion and the vessel permeability - vessel surface area product, and *v*_e_ reflects the extravascular extracellular volume fraction [15]. In DW-MRI, the apperant diffusion coefficient (ADC) is estimated. This parameter has been shown to reflect cell density and to be sensitive to necrotic tissue in untreated tumors [16, 17]. Reductions in *K*^trans^ or *K*^trans^ related parameters have been reported in most studies evaluating the effect of antiangiogetic treatment with DCE-MRI [14, 18], whereas both reductions and increases in ADC have been reported in studies evaluating the effect of antiangiogetic treatment with DW-MRI [19, 20]. In most of these studies, high doses of antiangiogenic agents have been used, and intratumor heterogeneity in the treatment-induced effects has not been investigated.

We have previously shown that DCE-MRI and DW-MRI are sensitive to effects of sunitinib treatment in human melanoma xenografts [21]. Sunitinib is a tyrosine kinase inhibitor which targets several receptors including vascular endothelial growth factor receptors 1-3 (VEGFR-1, -2, and -3), platelet-derived growth factor receptors α-β (PDGFR-α and PDGFR-β), stem cell growth factor receptor (c-KIT), and fms-like tyrosine kinase receptor 3 (FLT 3) [7]. In the previous study, we used a relatively high sunitinib dose which reduced microvascular density, increased hypoxic fractions, and induced necrosis. Moreover, the effect of treatment was evaluated once in xenografts from one melanoma line. In the current study, we evaluated the effect of low dose bevacizumab treatment with the same MR-techniques. Bevacizumab is a humanized monoclonal antibody that targets VEGF-A, and thus inhibits the VEGF-A pathway specifically [22]. Xenografts from two melanoma lines with different VEGF-A expression were used, and the tumors were subjected to DCE-MRI and DW-MRI before the treatment started and twice during a 7-days treatment period. We report that low dose bevacizumab treatment reduced *K*^trans^ in the high VEGF-A expressing tumors, and that the effect was more pronounced in peripherial than in central tumor regions.

## Methods

### Mice and tumors

Adult (8–12 weeks of age) female BALB/c*-nu/nu* mice, bred at our research institute, were used as host animals for xenografted tumors. Animal care and experimental procedures were approved by the Institutional Committee on Research Animal Care and were performed in accordance with the Interdisciplinary Principles and Guidelines for the Use of Animals in Research, Marketing, and Education (New York Academy of Sciences, New York, NY, USA). The experiments were performed with tumors of the amelanotic human melanomas A-07 and R-18, established and characterized as described previously [23]. A-07 and R-18 cells were obtained from our frozen stock and were cultured in RPMI-1640 medium (25 mM HEPES and L-glutamine) supplemented with 13 % bovine calf serum, 250 mg/l penicillin, and 50 mg/l streptomycin. Approximately 3.5 × 10^5^ cells in 10 μl of Hanks’ balanced salt solution (HBSS) were inoculated intradermally in the hind leg by using a 100-μl Hamilton syringe.

### Bevacizumab treatment

Mice were given two intraperitoneal doses of 5 mg/kg bevacizumab (Avastin, F. Hoffman-La Roche, Basel, Switzerland) or vehicle (saline), with 3 days between the doses.

### Anesthesia

MRI was carried out with anesthetized mice. Mice were given 0.5 L/min O_2_ containing ~4.0 % Sevofluran (Baxter, Illinois, USA) during MRI. Respiration rate and body core temperature were monitored continuously by using an abdominal pressure sensitive probe and a rectal temperature probe (Small Animal Instruments, New York, USA). The body core temperature of the mice was kept at 37 °C by adjusting the hot air flow automatically, and the sevofluran dose was adjusted to maintain a stable respiration rate.

### MR scanner and coil

MRI was performed by using a Bruker Biospec 7.05 T bore magnet with a mouse quadrature volume coil (Bruker Biospin, Ettlingen, Germany). The tumors were positioned in the isocenter of the magnet and were imaged with axial slices covering the entire tumor volume.

### DCE-MRI

A fast spin echo pulse sequence (RARE) with varying repetition time (TR = 200, 400, 800, 1500, and 5000 ms), an echo time (TE) of 8.5 ms, an image matrix of 128 × 128, a field of view (FOV) of 3 × 3 cm^2^, a slice thickness of 0.7 mm, and a slice gap of 0.3 mm was applied to measure precontrast T_1_-values (T_10_-map). Gd-DOTA (Dotarem, Guerbet, Paris, France), diluted to a final concentration of 0.06 M, was administered in the tail vein of mice in a bolus dose of 5.0 ml/kg during a period of 5 s by using an automated infusion pump (Harvard Apparatus, Holliston, MA, USA). A 3-dimensional SPGR pulse sequence (3D-FLASH) with a TR of 10 ms, a TE of 2.07 ms, a flip angle (α) of 20°, an image matrix of 128 × 128 × 10, and a FOV of 3 × 3 × 1 cm^3^ was applied to produce T_1_-weighted images with a spatial resolution of 0.23 × 0.23 × 1.0 mm^3^, and a temporal resolution of 14.8 s. T_1_-weighthed images were recorded before Gd-DOTA injection, and every 14.8 s for 15 min after the injection (6 precontrast, and 59 postcontrast images). According to the theoretical equation for SPGR pulse sequences [24, 25],

$$ S = {S}_0\cdot \frac{sin\alpha \cdot \left(1-{e}^{-TR/{T}_1}\right)}{1- cos\alpha \cdot {e}^{-TR/{T}_1}}\cdot {e}^{-TE/{T}_2^{*}}\approx {S}_0\cdot \frac{sin\alpha \cdot \left(1-{e}^{-TR/{T}_1}\right)}{1- cos\alpha \cdot {e}^{-TR/{T}_1}} $$

where *S* is the signal intensity, and *S*_*0*_ is a constant depending on scanner gain and proton density. The approximation ${e}^{-TE/{T}_2^{*}}=1$ is valid when *T*_2_^*^ ≫ *TE*, which was verified to be the case in our experiments. Images of phantoms with different Gd-DOTA concentration showed that the signal intensities produced by the 3D-FLASH followed the theoretical equation, confirming that the pulse sequence was appropriate for measurement of contrast agent concentration (Additional file 1). In contrast, the 2-dimensional SPGR pulse sequences available on the 7.05 T Bruker scanner (2D-FLASH) produced signal intensities that deviated substantially from the theoretical equation, and were thus inappropriate for measurement of contrast agent concentration (Additional file 1). Concentration of Gd-DOTA was calculated from the T_1_-weighted images in three steps. First, the constant *S*_*0*_ was calculated for each voxel by using the precontrast images and the *T*_*10*_-map. Seccond, *T*_*1*_-values were calculated for the postcontrast images. Third, the changes in *T*_*1*_-values were converted to Gd-DOTA concentrations (*C*) by using the equation [25]:

$$ C\cdot {r}_{Gd- DOTA} = \frac{1}{T_1}-\frac{1}{T_{10}} $$

*r*_*Gd-DOTA*_ is the relaxivity of Gd-DOTA which was measured to be 3.70 mM^−1^s^−1^ for the 7.05 T Bruker scanner. The DCE-MRI series were analyzed on a voxel-by-voxel basis by using the pharmacokinetic model described by Tofts et al. [15], and the arterial input function of Benjaminsen et al. [26]:

$$ {C}_t(T)=\frac{K^{trans}}{\left(1-Hct\right)}\cdot {\displaystyle {\int}_0^T{C}_a}(t)\cdot {e}^{- \frac{K^{trans}\cdot \left(T-t\right)}{v_e}}\ dt, $$

where *C*_*t*_*(T)* is the Gd-DOTA concentration in the tumor tissue at time *T*, *K*^trans^ is the transfer rate constant, *Hct* is the hematocrit, *C*_*a*_*(t)* is the arterial input function, and *v*_e_ is the fractional distribution volume of Gd-DOTA. Numerical values of *K*^trans^ and *v*_e_ were determined for each voxel from the best curve fit. Unphysiological voxels (voxels with *v*_e_ > 1) were excluded from the analysis. The number of unphysiological voxels did not differ between untreated and bevacizumab-treated tumors and were ~ 5 % for A-07 tumors, and ~ 2 % R-18 tumors. Calculation of Gd-DOTA concentrations and pharmacokinetic modeling were done with in-house-made software developed in Matlab (MathWorks, Natick, MA, USA).

### DW-MRI

DW-MRI was carried out by applying a diffusion-weighted single-shot fast spin echo pulse sequence (RARE) with a TR of 1300 ms, a TE of 26 ms, an image matrix of 64 × 64, a FOV of 3 × 3 cm^2^, a slice thickness of 0.7 mm, and a slice gap of 0.3 mm. Four different diffusion-weightings with diffusion encoding constants (*b*) of 200, 400, 700 and 1000 s/mm^2^, a diffusion gradient duration of 7 ms, and a diffusion separation time of 14 ms were used. Diffusion sensitization gradients were applied in three orthogonal directions with the following physical gradient combinations: [1 0 0], [0 1 0], [0 0 1]. ADC maps were produced with in-house-made software developed in Matlab. Briefly, the directional diffusion images were averaged on a voxel-by-voxel basis to non-directional diffusion images. ADC values were calculated for each voxel by fitting signal intensities (*S*) to the mono-exponential model equation:

$$ \ln \left(S(b)\right)=-b\cdot \mathrm{A}\mathrm{D}\mathrm{C}+c $$

by using a linear least square fit algorithm. The signal decay of a large number of voxels was investigated to verify that the mono-exponential model gave good fits to the data. The fits generally had a correlation coefficient of 0.95–0.99. DW-MRI was performed before injection of contrast agent.

### Statistical analysis

Statistical comparisons of data were carried out by the Student’s t test when the data complied with the conditions of normality and equal variance. Under other conditions, comparisons were done by nonparametric analysis using the Mann–Whitney rank sum test. The Kolmogorov-Smirnov method was used to test for normality, and the Levene’s test was used to test for equal variance. Probability values of *P* < 0.05, determined from two-sided tests, were considered significant. The statistical analysis was performed by using the SigmaStat statistical software (SPSS Science, Chicago, IL, USA).

## Results

**Fig. 1:**
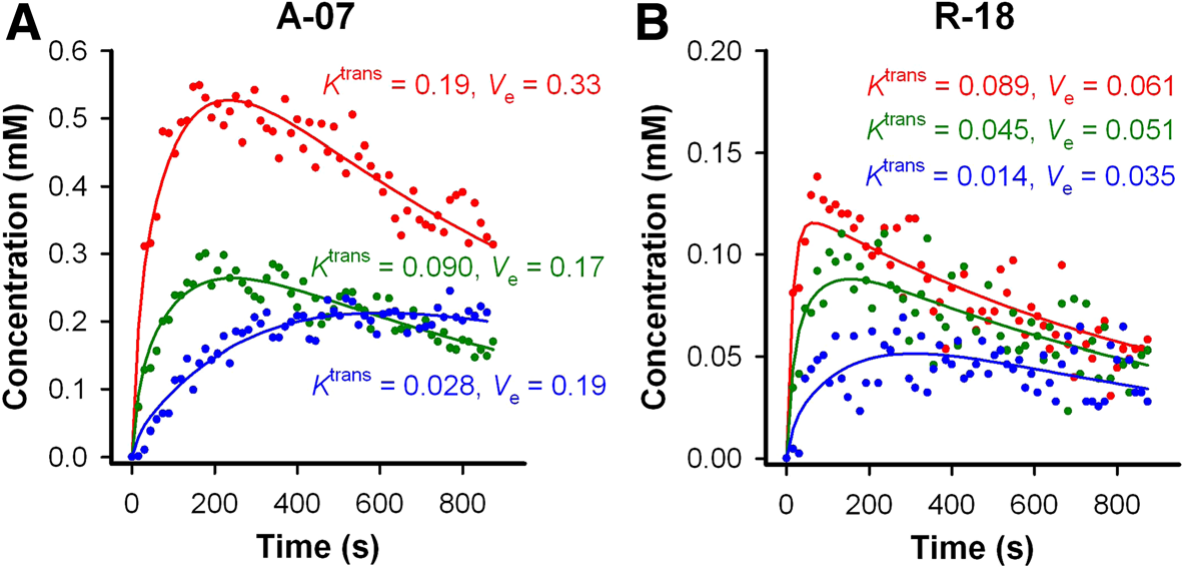
Uptake of Gd-DOTA in individual voxels. Plots of Gd-DOTA concentration versus time (*symbols*) and the corresponding pharmacokinetic model fits (*solid lines*) for individual voxels in a representative untreated A-07 tumor (**a**), and a representative untreated R-18 tumor (**b**). *K* ^trans^ and *v*_e_ values for the individual voxels were determined by pharmacokinetic analysis and are shown in the panels

**Fig. 2:**
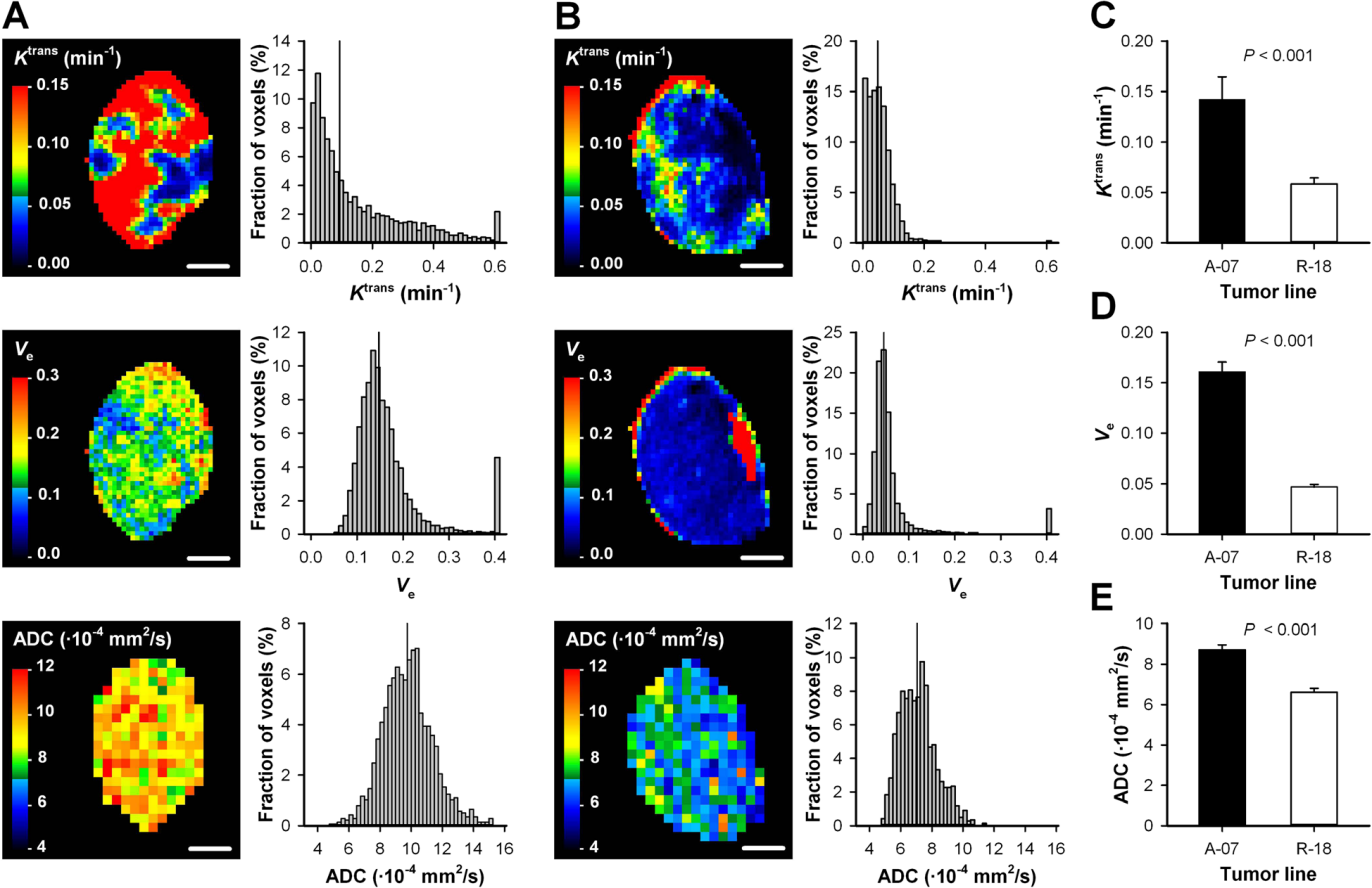
A-07 and R-18 tumors differed in *K* ^trans^, *v*_e_, and ADC. **a-b**, *K* ^trans^, *v*_e_, and ADC images, and *K* ^trans^, *v*_e_, and ADC frequency distributions of a representative untreated A-07 tumor (**a**), and a representative untreated R-18 tumor (**b**). The images refer to the central axial section of the tumor, whereas the frequency distributions are based on the individual voxel values of all the sections of the tumor. Color bars show *K* ^trans^, *v*_e_, or ADC scales, scale bars are 2 mm, and the vertical lines in the frequency distributions indicate median values. **c-e**, *K* ^trans^ (**c**), *v*_e_ (**d**), and ADC values (**e**) in untreated A-07 and R-18 tumors. Colums, means of 9-13 tumors, bars, SEM. Statistical comparisons of the data were carried out by the Student’s t test or the Mann–Whitney rank sum test. Untreated A-07 tumors showed significantly higher, *K* ^trans^, *v*_e_, and ADC values than untreated R-18 tumors (*P* < 0.001)

Untreated A-07 and R-18 tumors were subjected to DCE-MRI and DW-MRI to investigate whether the MR-techniques were sensitive to differences between these tumor lines. A-07 tumors generally showed higher uptake of Gd-DOTA than R-18 tumors, and the uptake differed substantially for individual voxels in both tumor lines. This is illustrated in Fig. 1 which shows plots of Gd-DOTA concentration versus time and the corresponding pharmacokinetic model fits for individual voxels in a representative A-07 and R-18 tumor. The signal-to-noise ratio was sufficiently high that well-defined pharmacokinetic model fits were produced for voxels with both high and low uptake of Gd-DOTA in both tumor lines. The *K*^trans^, *v*_e_, and ADC image and the *K*^trans^, *v*_e_, and ADC frequency distribution of a representative A-07 and R-18 tumor are presented in Fig. 2a-b. Untreated A-07 tumors showed significantly higher *K*^trans^, *v*_e_, and ADC values than untreated R-18 tumors (Fig. 2c-e; *P* < 0.001).

**Fig. 3:**
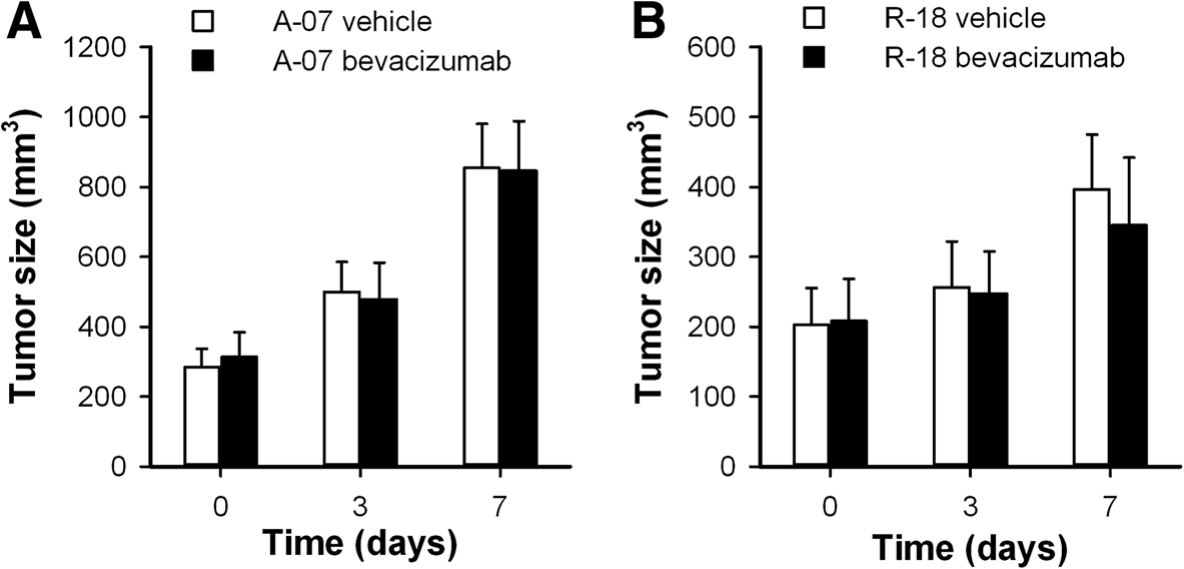
Low dose bevacizumab treatment did not affect tumor growth. Tumor size before treatment, and 3 and 7 days after the treatment started in untreated and bevacizumab treated A-07 (**a**) and R-18 (**b**) tumors. Colums, means of 4-7 tumors, bars, SEM. Statistical comparisons of the data were carried out by the Student’s t test or the Mann–Whitney rank sum test. Significant differences in tumor size were not found between untreated and bevacizumab-treated A-07 tumors (**a**; *P* > 0.05), or between untreated and bevacizumab-treated R-18 tumors (**b**; *P* > 0.05)

A-07 and R-18 tumors were divided in groups with matched tumor sizes to receive bevacizumab treatment or vehicle. The tumors were subjected to MRI before the treatment started (day 0), and twice during the treatment period (day 3 and day 7), allowing accurate measurement of tumor volume at these time points. All tumors grew during the 7-days treatment period, and the bevacizumab-treated tumors did not differ from the untreated tumors in size at any time point, regardless of whether A-07 (Fig. 3a; *P* > 0.05) or R-18 tumors (Fig. 3b; *P* > 0.05) were considered.

**Fig. 4:**
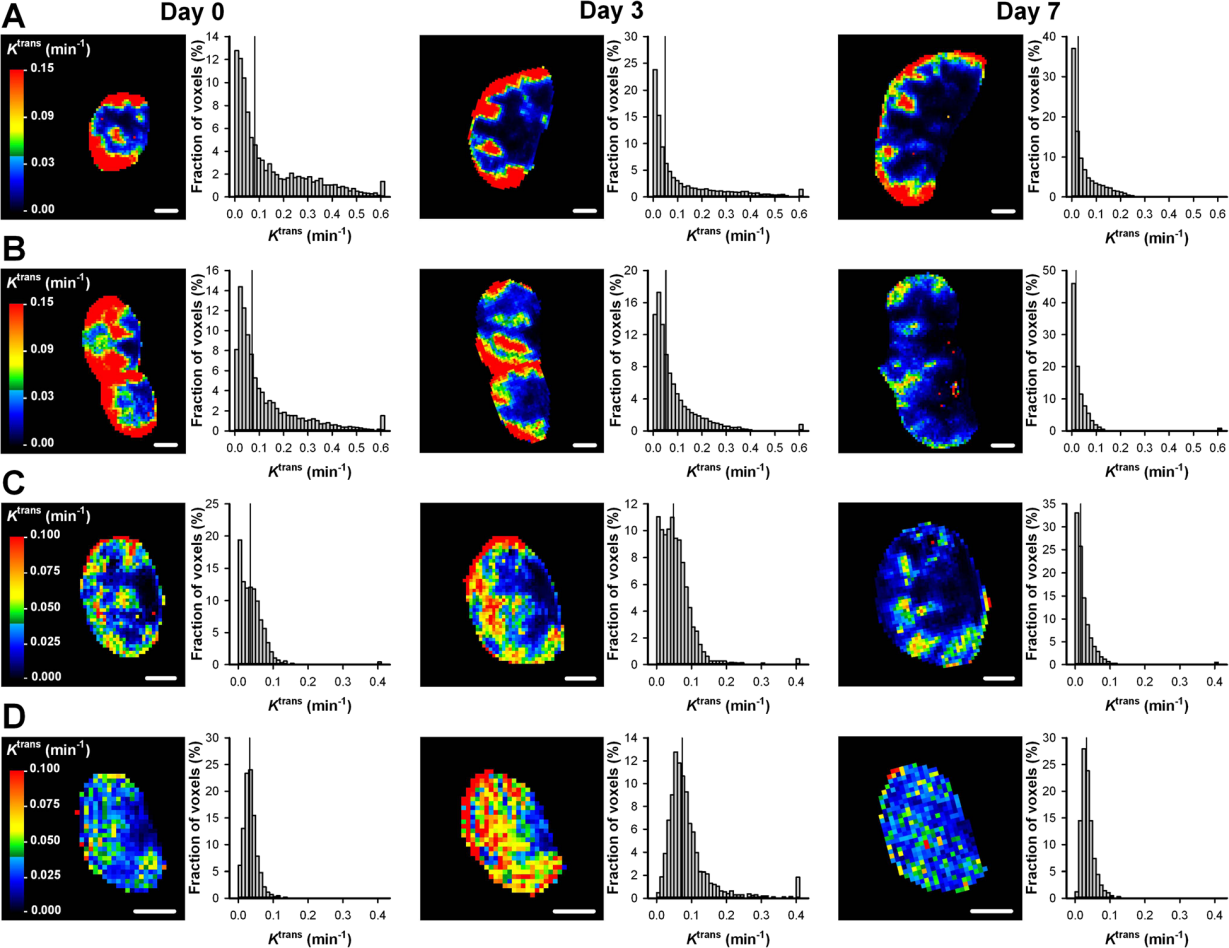
Low dose bevacizumab treatment reduced *K* ^trans^ in A-07 tumors. *K* ^trans^ images and *K* ^trans^ frequency distributions recorded before treatment, and 3 and 7 days after the treatment started. The panels show a representative untreated A-07 tumor (**a**), a representative bevacizumab-treated A-07 tumor (**b**), a representative untreated R-18 tumor (**c**), and a representative bevacizumab-treated R-18 tumor (**d**). The images refer to the central axial section of the tumor, whereas the frequency distributions are based on the individual voxel values of all the sections of the tumor. Color bars show *K* ^trans^ scales, scale bars are 2 mm, and the vertical lines in the frequency distributions indicate median values

**Fig. 5:**
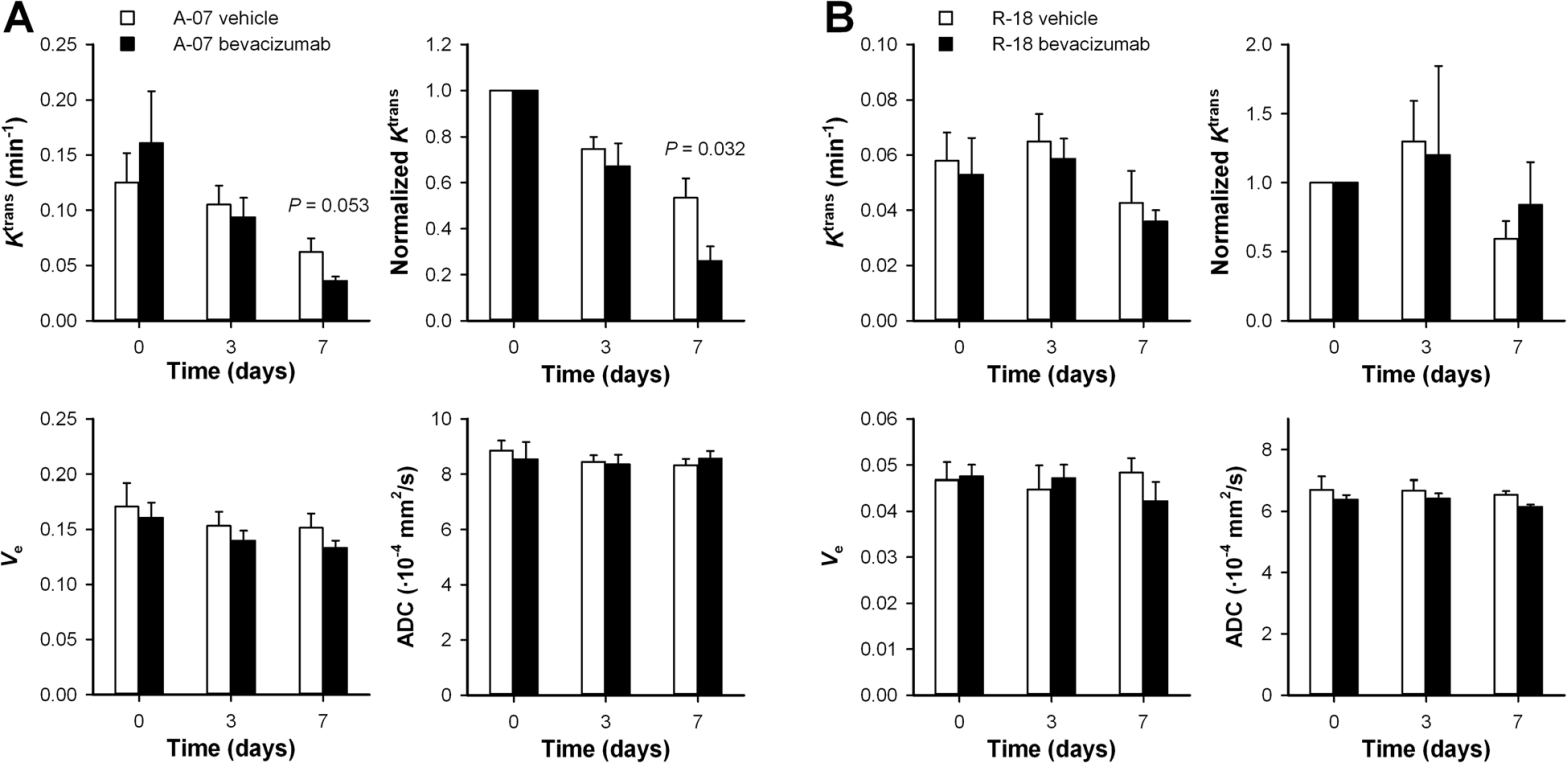
The effect of low dose bevacizumab treatment on *K* ^trans^, *v*_e_, and ADC. *K* ^trans^, normalized *K* ^trans^, *v*_e_, and ADC before treatment, and 3 and 7 days after the treatment started, in untreated and bevacizumab treated A-07 (**a**) and R-18 (**b**) tumors. Colums, means of 4-7 tumors, bars, SEM. Statistical comparisons of the data were carried out by the Student’s t test or the Mann–Whitney rank sum test. *P*-values are indicated in the panels where the statistical tests revealed significant or borderline significant differences between untreated and bevacizumab-treated tumors

A-07 and R-18 tumors differed in their response to low dose bevacizumab treatment. This is illustrated qualitatively in Fig. 4 which shows the *K*^trans^ images and the *K*^trans^ frequency distributions of a representative untreated A-07 tumor (Fig. 4a), a representative bevacizumab-treated A-07 tumor (Fig. 4b), a representative untreated R-18 tumor (Fig. 4c), and a representative bevacizumab-treated R-18 tumor (Fig. 4d). The images were recorded before treatment, and 3 and 7 days after the treatment start. Quantitative studies showed that *K*^trans^ values were significantly reduced during growth in A-07 tumors (Fig. 5a; day 7 *vs* day 0: *P* = 0.032 for untreated tumors, and *P* = 0.015 for bevacizumab-treated tumors). After the treatment period, *K*^trans^ values were lower in bevacizumab-treated than in untreated A-07 tumors, suggesting that the treatment reduced *K*^trans^ (Fig. 5a). This difference was borderline significant when the absolute values of *K*^trans^ were considered (*P* = 0.053) and significant when the *K*^trans^ values were normalized to the pretreatment values (*P* = 0.032). In R-18 tumors, changes in *K*^trans^ during growth were small, and significant differences between untreated and bevacizumab-treated tumors were not found, regardless of whether the absolute or normalized *K*^trans^ values were considered (Fig. 5b; *P* > 0.05). *v*_e_ and ADC values did not change during the treatment period for any of the tumor lines. Thus neither growth-induced nor treatment-induced changes in these parameters were observed (Fig. 5a-b; *P* > 0.05).

**Fig. 6:**
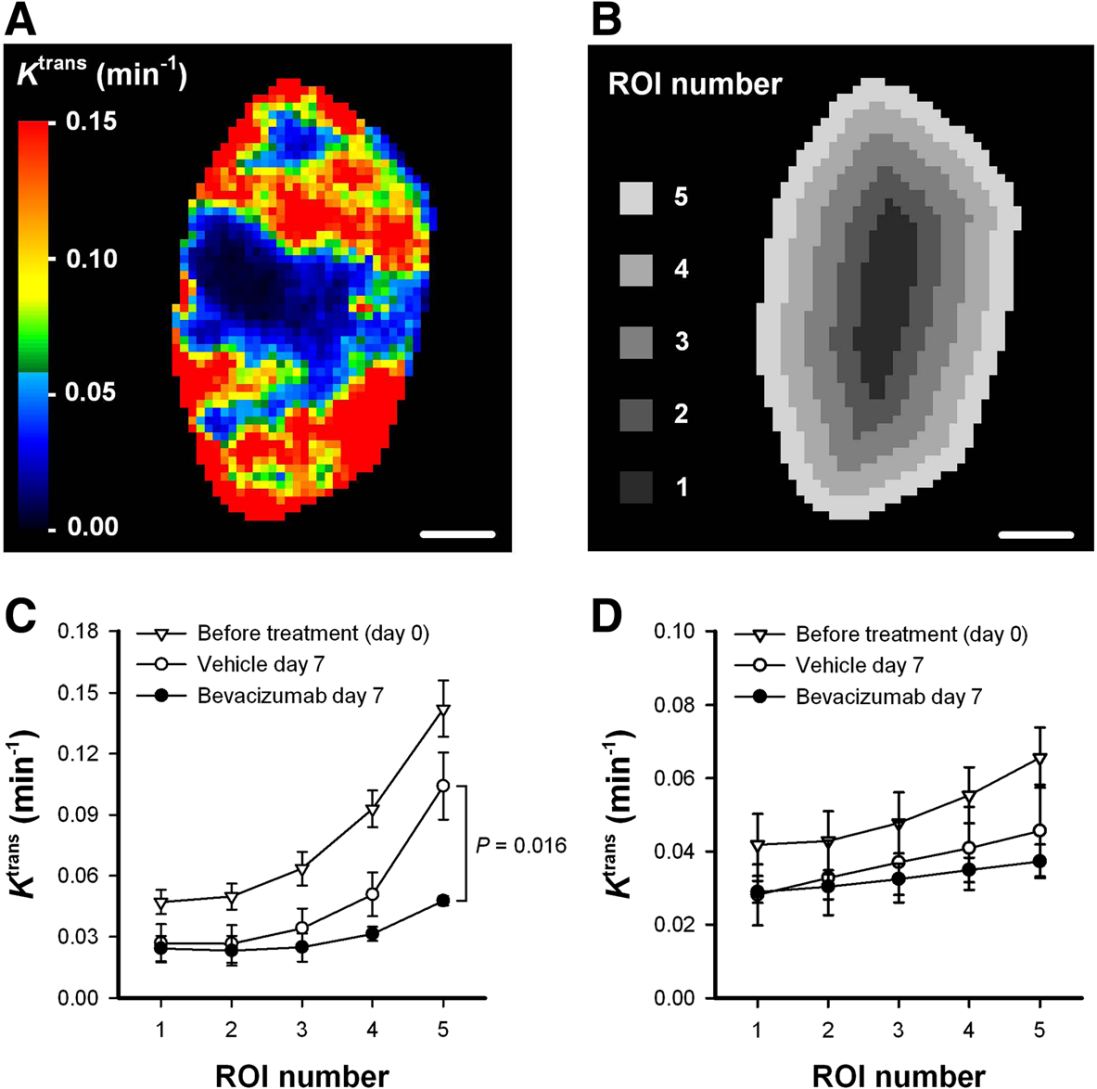
Intratumor heterogeneity in the effect of low dose bevacizumab treatment. **a-b**, *K* ^trans^ image of a representative untreated A-07 tumor (**a**), and image illustrating how the tumor was divided in 5 concentric circular ROIs (**b**). The circular ROIs are bounded by lines drawn at distances of nR/5 from the tumor center, where R is tumor radius and n is ROI number. Color bar shows *K* ^trans^ scale, scale bars are 2 mm. **c-d**, *K* ^trans^ in 5 concentric circular ROIs before treatment, and in untreated and bevacizumab-treated tumors 7 days after the treatment started. The graphs refer to A-07 (**c**) and R-18 (**d**) tumors. Symbols, means of 4–9 tumors, bars, SEM. Statistical comparisons of the data were carried out by the Student’s t test or the Mann–Whitney rank sum test. After the treatment period (day 7), differences in *K* ^trans^ values between untreated and bevacizumab-treated A-07 tumors were not significant in ROI 1–3 (*P* > 0.05), were borderline significant in ROI 4 (*P* = 0.063), and were significant in ROI 5 (*P* = 0.016). For R-18 tumors, significant differences between untreated and bevacizumab-treated tumors were not found in any ROI (P > 0.05)

To investigate intratumor heterogeneity in treatment effects, tumor images were divided in 5 concentric regions of interest (ROIs) as illustrated in Fig. 6a-b. Before the treatment period, A-07 tumors showed radial heterogeneity in *K*^trans^, i.e., *K*^trans^ values were low in the central ROIs and increased gradually towards the tumor periphery (Fig. 6c; day 0, ROI 1 vs ROI 5: *P* < 0.001). In untreated A-07 tumors, *K*^trans^ was similarly reduced in all ROIs during the treatment period, and thus tumor growth did not alter the radial heterogeneity (Fig. 6c; day 0 vs vehicle day 7). Compared with untreated tumors, bevacizumab-treated A-07 tumors showed similar *K*^trans^ values in the central ROIs and significantly lower *K*^trans^ values in the tumor periphery after the treatment period (Fig. 6c; vehicle day 7 vs bevacizumab day 7: *P* > 0.05 for central ROIs, and *P* = 0.016 for peripherial ROI). This implies that the treatment was more effective for peripherial than for central tumor regions, and consequently the treatment altered the radial heterogeneity in A-07 tumors. Radial heterogeneity in *K*^trans^ was also found in R-18 tumors before the treatment period (Fig. 6d; day 0, ROI 1 vs ROI 5: *P* = 0.048). After the treatment period, bevacizumab-treated R-18 tumors did not differ from untreated R-18 tumors in any ROI, indicating that the treatment did not affect any region in these tumors (Fig. 6d; vehicle day 7 vs bevacizumab day 7: *P* > 0.05).

## Discussion

A-07 and R-18 melanoma xenografts were used as tumor models in the current study. We have previously shown that A-07 and R-18 cells differ substantially in the expression and secretion of VEGF-A, and that A-07 tumors have higher microvascular density, higher blood perfusion, and lower cell density than R-18 tumors [27–29]. In the current study, we demonstrate that these tumor lines also differ in the MR-derived parameters *K*^trans^, *v*_e_, and ADC, and we demonstrate that the tumor lines differ in their response to low dose bevacizumab treatment.

*K*^trans^ generally reflects blood perfusion and the vessel permeability - vessel surface area product [15]. However, in tumors with a high and uniform vessel permeability, the uptake of small-size contrast agents like Gd-DOTA is not limited by the vessel permeability, and *K*^trans^ reflects blood perfusion [30]. We have previously shown that A-07 and R-18 tumors show high and similar permeability for macromolecules, and that *K*^trans^ reflects blood perfusion in these tumor lines [31, 32]. Consequently, the difference in *K*^trans^ values between A-07 and R-18 tumors reported here probably reflected a difference in blood perfusion between the tumor lines. *v*_e_ reflects the extravascular extracellular volume fraction which is inversely correlated to the cell density [15], and ADC has been shown to reflect cell density and to be sensitive to necrosis [16, 17]. Untreated A-07 and R-18 tumors show insignificant necrotic fractions but differ substantially in cell density [29]. The difference in *v*_e_ and ADC values between A-07 and R-18 tumors thus probably reflected a difference in cell density between the tumor lines.

We have previously shown that sunitinib treatment reduces microvascular density and *K*^trans^ values in A-07 tumors, suggesting that the sunitinib-induced reduction in *K*^trans^ reflected reduced blood perfusion in that study [21]. It is highly likely that the treatment-induced reduction in *K*^trans^ observed in the current study also reflected reduced blood perfusion, because both sunitinib and bevacizumab treatment inhibit the VEGF-A pathway [7]. Inhibition of the VEGF-A pathway has also been shown to reduce vessel permeability in experimental tumors [5], and consequently, the bevacizumab-induced reduction in *K*^trans^ reported here may also have been influenced by reduced vessel permeability. The bevacizumab treatment did not affect *v*_e_ and ADC values in any of the tumor lines, implying that the treatment did not change cell density and did not induce necrosis. This observation confirms that the bevacizumab dose was low. In contrast, increased ADC values reflecting induction of tumor necrosis have been observed after sunitinib treatment in A-07 tumors [21].

The different effect of low dose bevacizumab treatment between A-07 and R-18 tumors was probably a consequence of a difference in the rate of VEGF-A induced angiogenesis. Thus A-07 tumors show high VEGF-A expression, high microvascular density, and high pretreatment *K*^trans^ values, and respond to bevacizumab treatment with reduced *K*^trans^ values, whereas R-18 tumors show low VEGF-A expression, low microvascular density, low pretreatment *K*^trans^ values, and no change in *K*^trans^ values after bevacizumab treatment.

In A-07 tumors, bevacizumab treatment reduced *K*^trans^ in peripherial regions with high pretreatment *K*^trans^ values and had little or no effect in central regions with low pretreatment *K*^trans^ values. We have previously demonstrated that untreated A-07 tumors show radial heterogeneity in several vascular parameters including microvascular density and blood perfusion [26, 33], suggesting that these tumors show similar heterogeneity in the rate of angiogenesis. These observations suggest that bevacizumab treatment was most effective in tumor regions with a high angiogenic rate. This suggestion is consistent with several studies reporting that antiangiogenic agents selectively removes immature blood vessels, because the fraction of immature blood vessels is expected to be high in tumor regions with high angiogenic rates [6, 12, 34].

In most studies evaluating the effect of antiangiogenic treatment with MR techniques, intratumor heterogeneity in the treatment effect has not been investigated [35]. Our study demonstrates that the effect of antiangiogenic treatment may be highly heterogeneous within tumors, and that careful monitoring of intratumor heterogeneities may provide mechanistic information about treatment effects and may identify poorly responding tumor regions. Detection of poorly responding regions can be important in a therapeutic setting because these regions may repopulate the tumor even if the treatment completely eradicates the tumor mass in other regions. If the effect of antiangiogenic treatment is evaluated with average parameters, poorly responding tumor regions may be overlooked. In addition, our study suggests that treatment-induced effects may be separated from growth-induced effects by evaluating changes in intratumor heterogeneities.

Treatment-induced reductions in tumor size generally occur late after antiangiogenic treatment [8]. However, if non-responding patients could be identified shortly after treatment initiation, any ineffective treatment could be stopped to avoid toxicity, and other treatments could be considered. In the current study, low dose bevacizumab reduced *K*^trans^ without affecting tumor growth, suggesting that DCE-MRI may be used to identify patients that respond to low dose bevacizumab treatment before treatment-induced reductions in tumor size can be detected.

It has been suggested that antiangiogenic agents including bevacizumab can selectively remove immature blood vessels, increase tumor perfusion, and increase oxygenation [5, 12, 13]. These effects have been labeled vascular normalization and have been reported to occur within a limited time period [36]. Vascular normalization may be reversed if the treatment is stopped, and tumors may switch to other angiogenesis pathways during treatment and become resistant to the antiangiogenic agents. Moreover, the beneficial effects of vascular normalization may be balanced by severe vascular regression after prolonged exposure to antiangiogenic agents, or if the dose of the antiangiogenic agent is too large [36]. Appropriate timing and low doses are thus required to induce vascular normalization. It has also been demonstrated that inhibition of the VEGF-A pathway fails to normalize tumor vasculature and induces hypoxia in some preclinical tumors, suggesting that vascular normalization cannot be induced in all tumor models [6, 34]. In the current study, low dose bevacizumab treatment did not increase blood perfusion in A-07 and R-18 human melanoma xenografts. It is unlikely that the lack of increased blood perfusion was due to inadequate observation time points or to too large bevacizumab dose, because both similar and higher bevacizumab doses have been shown to increase blood perfusion and oxygenation at similar time points in several tumor models, including human breast carcinoma xenografts, human ovarian carcinoma xenografts, human neuroblastoma xenografts, murine melanoma, and murine breast carcinoma [5, 12 37]. The effect of low dose bevacizumab treatment reported here is similar to our previous experience with sunitinib treatment. In those studies, sunitinib treatment did not improve vascular function but induced hypoxia in A-07 and R-18 tumors [11, 21]. Taken together, our current and previous studies suggest that inhibition of the VEGF-A pathway does not induce vascular normalization in these melanoma lines.

In tumors where antiangiogenic treatment induces hypoxia, neoadjuvant antiangiogenic therapy is expected to reduce the effect of radiation and chemotherapy [9, 10]. In contrast, neoadjuvant bevacizumab treatment has been shown to enhance the effect of radiation and chemotherapy in preclinical tumors where bevacizumab normalizes the vasculature and the microenvironment [5, 12]. We have previously shown that DCE-MRI and DW-MRI can be used to identify tumors where antiangiogenic treatment does not normalize the microenvironment [21]. In that study, sunitinib treatment reduced *K*^trans^ values and increased ADC values reflecting reduced perfusion and induction of tumor necrosis. The current study suggests that DCE-MRI can be used to identify such tumors, also when the treatment does not induce necrosis. These tumors respond to antiangiogenic treatment with reduced *K*^trans^ values and no change in ADC values. Others have reported that vascular normalization results in increased *K*^trans^ values and reduced ADC values [19 38]. Taken together, these studies suggest that DCE-MRI and DW-MRI may be used to monitor the effect of antiangiogenic treatment to detect vascular normalization, and to identify tumors where such treatment does not induce vascular normalization. Importantly, the MR-techniques are able to identify tumors where antiangiogenic treatment does not normalize the vasculature also when the treatment effect is small and tumor necrosis is not induced.

## Conclusion

A-07 and R-18 tumors differed in the response to low dose bevacizumab treatment, and the response was associated with the rate of VEGF-A induced angiogenesis. Effects of low dose bevacizumab treatment were detected by DCE-MRI before tumor growth was affected. Our study suggests that DCE-MRI may be used to identify tumors where antiangiogenic treatment does not induce vascular normalization, also when the treatment does not induce necrosis. Moreover, the effect of low dose bevacizumab treatment was highly heterogeneous within A-07 tumors. Our study demonstrates that careful monitoring of intratumor heterogeneity may identify poorly responding tumor regions, may provide mechanistic information about the treatment effect, and may be used to differentiate treatment-induced from growth-induced effects in tumors similar to A-07 tumors.

## Additional file

Additional file 1:
**Signal intensities in phantoms measured with** **2D-FLASH** **and** **3D-FLASH.** (PDF 73 kb)
