# Supplementary material for: Early effects of low dose bevacizumab treatment assessed by magnetic resonance imaging
Source: BMC Cancer. 2015 Nov 14;15:900. doi: 10.1186/s12885-015-1918-1 (PMC4647606; doi:10.1186/s12885-015-1918-1)
Supplement: Additional file 1: — Signal intensities in phantoms measured with 2D-FLASH and 3D-FLASH. (PDF 73 kb) [file 12885_2015_1918_MOESM1_ESM.pdf]

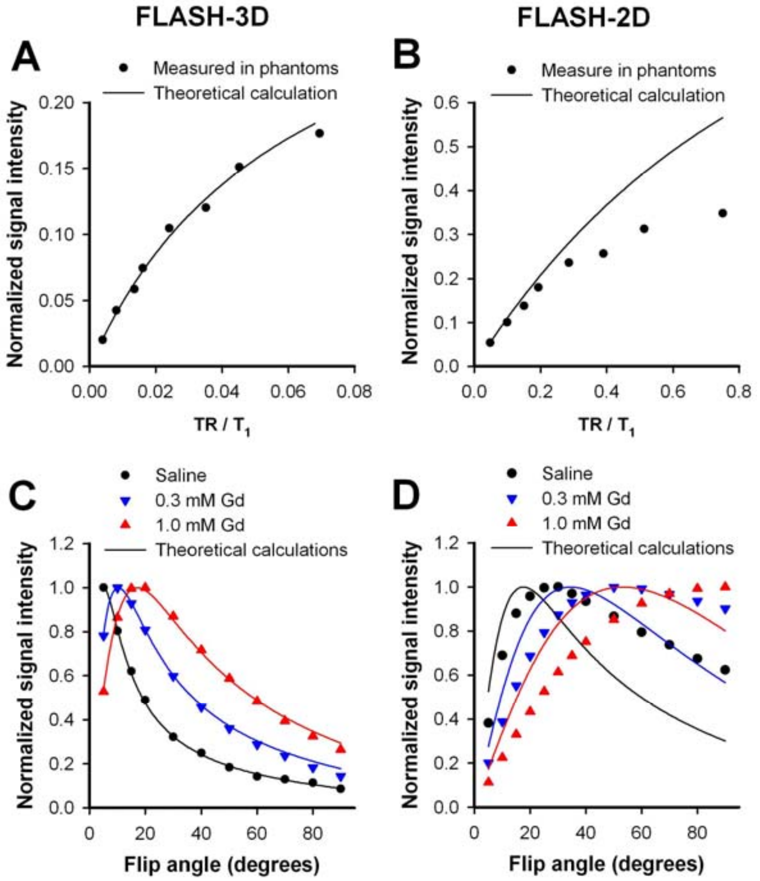

Additional file 1. Signal intensities in phantoms measured with 2D-FLASH and 3D-FLASH.

8 phantoms with saline and different concentrations of Gd-DOTA (0, 0.1, 0.2, 0.3, 0.5, 0.7, 1.0, and 1.5 mM) were imaged with a two- and three-dimensional SPGR pulse sequence (2D-FLASH and 3D-FLASH). Signal intensities measured with 3D-FLASH followed the theoretical equation for SPGR pulse sequences, whereas signal intensities measured with 2D-FLASH deviated substantially from the theoretical equation. **A-B**, normalized signal intensity versus TR/T1 measured in images produced with 3D-FLASH (**A**) or 2D-FLASH (**B**). Symbols refer to median values in individual phantoms and the solid line refers to theoretical calculations made by using the theoretical equation for SPGR pulse sequences. **C-D**, normalized signal intensity versus flip angle measured in images produced with 3D-FLASH (**C**) or 2D-FLASH (**D**). Symbols refer to median values obtained with different flip angles in three of the phantoms (black circles, saline; blue triangles, 0.3 mM Gd-DOTA; red triangles, 1.0 mM Gd-DOTA), and the solid lines refer to theoretical calculations made by using the theoretical equation for SPGR pulse sequences. The 3D-FLASH was performed with a TR of 10 ms, a TE of 2.07 ms, an image matrix of 128 × 128 × 10, a FOV of 3 × 3 × 1 cm^3, and a flip angle of 20° (**A**), or varying flip angles (5- 90°; **C**). The 2D-FLASH was performed with a TR of 100 ms, a TE of 1.86 ms, an image matrix of 128 × 128, FOV = 3 × 3 cm, a slice thickness of 1.0 mm, and a flip angle of 80° (**B**), or varying flip angles (5- 90°; **D**)
